# Supplementary material for: The Systems Biology Research Tool: evolvable open-source software
Source: BMC Syst Biol. 2008 Jun 29;2:55. doi: 10.1186/1752-0509-2-55 (PMC2446383; doi:10.1186/1752-0509-2-55)
Supplement: Additional file 1 — SBRT Archive. An archive of the current version of the Systems Biology Research Tool. [file 1752-0509-2-55-S1.zip › sbrt-1.4.0/doc/developers_guide/api/sbrt/shell/text/package-summary.html]

sbrt.shell.text


|  |  |  |
| --- | --- | --- |
| **Overview** \| **Package** \| Class \| **Use** \| **Tree** \| **Deprecated** \| **Index** \| **Help** | |  |
| **PREV PACKAGE**   **NEXT PACKAGE** | **FRAMES**    **NO FRAMES**     **All Classes** |  |


---

## Package sbrt.shell.text

Provides classes and interfaces for defining formats for basic objects.

**See:**
  
          **Description**

| **Interface Summary** | |
| --- | --- |
| **AppInputFileLineFormat** | This interface is used to represent the format of lines in process input files. |
| **BooleanFormat** | This interface is used to represent a format for boolean values. |
| **CollectionFormat<E>** | This interface is used to represent collection formats. |
| **DoubleFormat** | This interface is used to represent a format for double precision numbers. |
| **Format** | This interface is used to represent formats. |
| **Formatter<T>** | This interface is used to represent *formatters*. |
| **IntegerFormat** | This interface is used to represent a format for integers. |
| **LinkedHashSetFormat<E>** | This interface is used to represent `LinkedHashSet` formats. |
| **ListFormat<E>** | This interface is used to represent list formats. |
| **LongFormat** | This interface is used to represent a format for longs. |
| **MapEntryFormat<K,V>** | This interface is used to represent formats for map entries. |
| **MapFormat<K,V>** | This interface is used to represent formats for maps. |
| **Parser<T>** | This interface is used to represent *parsers*. |
| **PercentFormat** | This interface is used to represent a format for percentages. |
| **SimpleFormat<T>** | This interface is used to represent simple formats. |

| **Class Summary** | |
| --- | --- |
| **AbstractLinkedHashSetFormat<E>** | This abstract class is a skeleton implementation of `LinkedHashSetFormat`. |
| **AbstractListFormat<E>** | This abstract class is a skeleton implementation of `ListFormat`. |
| **AbstractMapEntryFormat<K,V>** | This abstract class is a skeleton implementation of `MapEntryFormat`. |
| **AbstractMapFormat<K,V>** | This abstract class is a skeleton implementation of `MapFormat`. |
| **AmpersandSetFormat<E>** | This class is used to define a format for ampersand-delimited sets. |
| **AppInputFileLineFormatVersion1** | This class is a concrete implementation of `AppInputFileLineFormat`. |
| **BoolRelFormatV1<E>** | This interface is used to represent boolean relationship formats. |
| **ColonMapEntryFormatV1<K,V>** | This class is a concrete implementation of `MapEntryFormat`. |
| **ColonMapFormatV1<K,V>** | This class is a concrete implementation of `MapFormat`. |
| **DoubleFormatV1** | This class is a concrete implementation of `DoubleFormat`. |
| **DoubleFormatV2** | This class is a concrete implementation of `DoubleFormat`. |
| **EqualsMapEntryFormatV1<K,V>** | This class is a concrete implementation of `MapEntryFormat`. |
| **EqualsMapFormatV1<K,V>** | This class is a concrete implementation of `MapFormat`. |
| **FileFormatFormat** | This class is used to represent formats for types of file formats. |
| **IntegerFormatV1** | This class is a concrete implementation of `IntegerFormat`. |
| **LongFormatV1** | This class is a concrete implementation of `LongFormat`. |
| **NonEmptyStringFormat** | This class is used to define a format for `String` objects. |
| **PercentFormatVersion1** | This class is a concrete implementation of `PercentFormat`. |
| **PipeListFormat<E>** | This class is used to define a format for pipe-delimited lists. |
| **PipeSetFormat<E>** | This class is used to define a format for pipe-delimited sets. |
| **SimpleListFormat<E>** | This interface is used to wrap `ListFormat` objects as `SimpleFormat` objects. |
| **SimpleSetFormat<E>** | This interface is used to wrap `ListFormat` objects as `SimpleFormat` objects. |
| **SpecialChars** | This class contains a set of characters that could be considered as *special* in some situations. |
| **StringFormat** | This class is used to define a format for `String` objects. |
| **TabListFormat<E>** | This class is used to define a format for tab-delimited lists. |
| **TabSetFormat<E>** | This class is used to define a format for tab-delimited sets. |
| **TrueFalseFormat** | This class is used to define a format for true/false values. |
| **WhitespaceListFormat<E>** | This class is used to define a format for whitespace-delimited lists. |
| **WhitespaceSetFormat<E>** | This class is used to define a format for whitespace-delimited sets. |
| **YesNoFormat** | This class is used to define a format for yes/no values. |

| **Exception Summary** | |
| --- | --- |
| **FormatException** | This exception type is used to indicate a formatting error has ocurred. |

## Package sbrt.shell.text Description

Provides classes and interfaces for defining formats for basic objects.

---


|  |  |  |
| --- | --- | --- |
| **Overview** \| **Package** \| Class \| **Use** \| **Tree** \| **Deprecated** \| **Index** \| **Help** | |  |
| **PREV PACKAGE**   **NEXT PACKAGE** | **FRAMES**    **NO FRAMES**     **All Classes** |  |


---
